# Post-training TMS abolishes performance improvement and releases future learning from interference

**DOI:** 10.1038/s42003-019-0566-4

**Published:** 2019-08-27

**Authors:** Ji Won Bang, Diana Milton, Yuka Sasaki, Takeo Watanabe, Dobromir Rahnev

**Affiliations:** 1 School of Psychology, Georgia Institute of Technology, Atlanta, GA 30332 USA; 2 Department of Ophthalmology, School of Medicine, New York University, New York, NY 10016 USA; 3 Department of Cognitive, Linguistic, and Psychological Sciences, Brown University, Providence, RI 02912 USA

**Keywords:** Perception, Consolidation

## Abstract

The period immediately after the offset of visual training is thought to be critical for memory consolidation. Nevertheless, we still lack direct evidence for the causal role of this period to perceptual learning of either previously or subsequently trained material. To address these issues, we had human subjects complete two consecutive trainings with different tasks (detecting different Gabor orientations). We applied continuous theta burst stimulation (cTBS) to either the visual cortex or a control site (vertex) immediately after the offset of the first training. In the vertex cTBS condition, subjects showed improvement on the first task but not on the second task, suggesting the presence of anterograde interference. Critically, cTBS to the visual cortex abolished the performance improvement on the first task and released the second training from the anterograde interference. These results provide causal evidence for a role of the immediate post-training period in the consolidation of perceptual learning.

## Abstract (web summary)

Ji Won Bang et al. test the importance of the immediate post-visual training period for memory consolidation in human subjects. They apply continuous theta burst stimulation (cTBS) directly following a learning task and show that this does affect memory consolidation, and that this effect relies on the visual cortex.

## Introduction

Learning new skills is one of the critical challenges that the brain needs to solve. Mounting evidence suggests that new learning undergoes a period of consolidation during offline states following initial training^1^. Such memory consolidation has been observed in a variety of species and can occur both during awake periods and sleep^2–4^.

Within the domain of motor learning, successful memory consolidation has been shown to depend on the processes occurring immediately after the offset of training. Early evidence came from studies that used a second motor training to abolish the performance improvement brought about by the first training^5,6^. More direct evidence has come from studies that employed transcranial magnetic stimulation (TMS) to disrupt the neural processes that take place after training offset. Several such studies demonstrated that TMS to the primary motor cortex after the offset of training can also abolish the performance improvement of the preceding training^7–9^, providing strong evidence for the causal role of the neural processes during the post training period in the consolidation of motor learning.

Since motor and visual learning share many common characteristics^10^, one may expect that the immediate post training period is critical in visual perceptual learning too. However, current evidence for the role of this period in the consolidation of perceptual learning comes only from behavioral studies. For example, performance improvement on one task can be abolished when the post training consolidation is disrupted by the learning of a second task. Such effects have been demonstrated in Vernier acuity^11^, texture discrimination^12^, and orientation detection tasks^13^. Nevertheless, direct causal evidence for the role of post training neural processes in the consolidation of visual learning is lacking.

What is more, neither the motor nor the perceptual learning literature has addressed the issue of the functional role of the immediate post training for future learning. For example, it is well known that training on one task can create interference with a subsequent training on a different task, a phenomenon known as “anterograde interference”^14–17^. It is likely that the neural processes occurring during the interval between the two trainings mediate this interference^17^ but this link is yet to be causally established.

The goal of the current study, therefore, was to both causally establish the role of the immediate post training period to visual perceptual learning and characterize the functional role of this period for future learning. We used continuous theta burst stimulation (cTBS^18^) to directly disrupt the neural processes in the early visual cortex immediately after the end of visual training. The early visual cortex was targeted because this area shows changes after visual training^2,13,19–26^, suggesting that it is involved in visual perceptual learning. A second training was presented 1 h after the offset of the first. This period was chosen based on two competing considerations: first, the intertraining interval had to be long enough for the effects of cTBS to largely dissipate, and, second, the intertraining interval had to be short enough for anterograde interference to take place. We previously found interference when the second training was given 30 min but not 3.5 h after the first training^13,27^. At the same time, the effects of cTBS are thought to dissipate around 1 h after stimulation^18^. Therefore, we chose to present the two trainings exactly 1 h apart in order to minimize any cTBS effects on the second training while also maximizing the chance of observing anterograde interference.

To anticipate, we found that applying cTBS to the early visual cortex abolished the performance improvement on the preceding task. Furthermore, this abolishment resulted in greater improvement for the second training performed 1 h later. These findings demonstrate that the immediate post training neural processes within the early visual cortex causally contribute to the consolidation process of visual learning and disrupting them allows future learning to take place.

## Results

### Task and experimental procedure overview

**Fig. 1:**
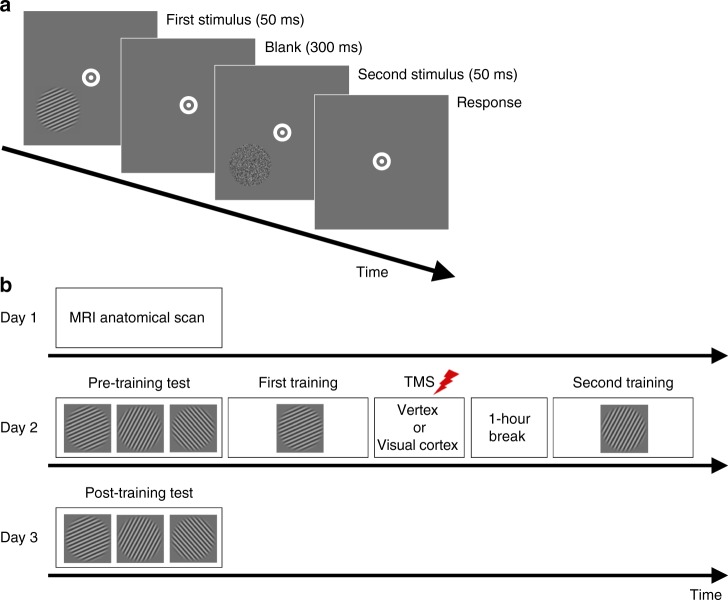
Task and experimental procedure. **a** Subjects performed a 2-interval forced-choice (2IFC) orientation detection task. Two stimuli—a target consisting of a Gabor patch embedded in noise and a nontarget consisting of pure noise—were presented in quick temporal succession. Subjects reported which interval contained the target. The Gabor patch was always presented either in the lower-left or lower-right quadrant (counter-balanced between subjects). **b** The experiment consisted of 3 days. An MRI anatomical scan was conducted on day 1 (only for subjects who subsequently received stimulation to their visual cortex). Day 2 began with a baseline pre training test for each of three stimulus orientations (10°, 70°, and 130°). Subjects were then trained on one randomly chosen orientation and received continuous theta burst stimulation (cTBS) within ~2–3 min from the training offset. For each subject, cTBS was delivered either to the early visual cortex or vertex (control site). For those who were assigned to the visual cortex condition, cTBS was applied to the part of the early visual cortex corresponding to the trained quadrant. After a 1-h break, subjects completed a second training on a different, randomly chosen orientation. On day 3, a post training test assessed how much learning took place for each orientation (trained first, trained second, and untrained)

We investigated whether directly disrupting the post training neural processes in the visual cortex has a causal effect on visual perceptual learning. Subjects engaged in two consecutive training sessions. In each session, subjects learned to detect a low contrast Gabor patch of a particular orientation. For each subject, the Gabor patches were always presented in the lower-left or lower-right quadrant (counter-balanced between subjects). In a first group of subjects, we applied cTBS to the dorsal segment of the contralateral early visual cortex immediately after the offset of the first training (Fig. 1). In a control group of subjects, cTBS was instead delivered to the vertex. We examined the effects of visual cortex cTBS on the learning for the orientation trained immediately before cTBS application, as well as for the second Gabor orientation trained 1 h later. Learning was quantified as the percent performance improvement from the pre- to post-training tests.

### Matched performance on the pre training tests

We first confirmed that the performance on the pre training tests was comparable across the two sites of stimulation (visual cortex vs. vertex) and the three training categories (trained first vs. trained second vs. untrained). We performed a two-way mixed measures ANOVA on the performance during the pre training tests with factors cTBS site (between-subjects factor) and training category (within-subjects factor). We found no main effect of cTBS site (*F*(1,23) = 0.741, *P* = 0.398, *η*^2^ = 0.031), no main effect of training category (*F*(2,46) = 0.684, Huynh–Feldt correction, epsilon = 0.678, *P* = 0.457, *η*^2^ = 0.029), and no interaction between cTBS site and training category (*F*(2,46) = 0.303, Huynh–Feldt correction, epsilon = 0.678, *P* = 0.655, *η*^2^ = 0.013). These results indicate that the initial, pre training performance was comparable across the different conditions of our experiment.

### Effect of cTBS on performance improvement for each training

**Fig. 2:**
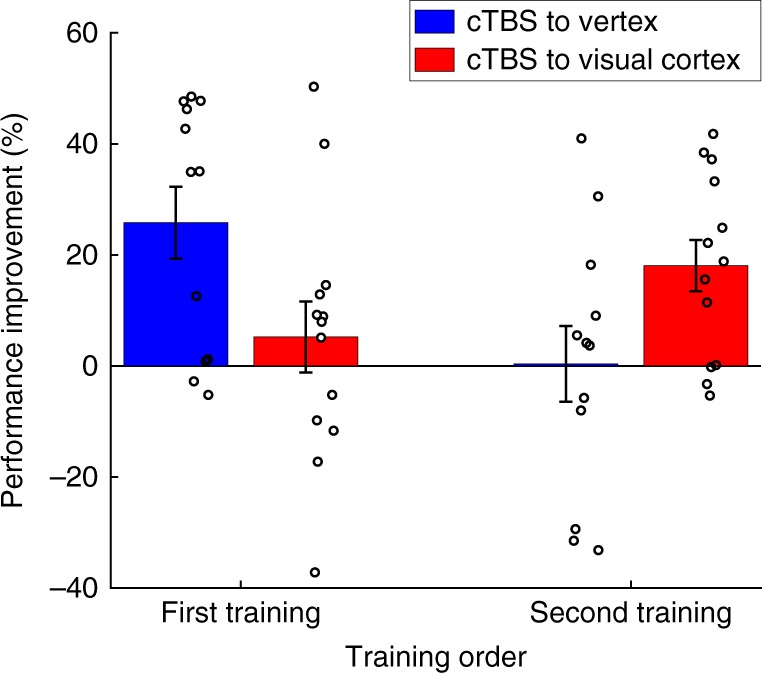
Learning as a function of training order and cTBS site. We observed an interaction between cTBS site and order of training. Specifically, cTBS to the visual cortex abolished performance improvement for the first training but increased it for the second training. In other words, cTBS to the visual cortex disrupted the pre stimulation learning and released the second learning from anterograde interference. Vertex served as a control site. Error bars represent s.e.m. *N* = 13 for the visual cortex group. *N* = 12 for the vertex group

We then examined the effect of cTBS on perceptual learning. We conducted a two-way mixed measures ANOVA on the performance improvement from the pre- to the post-training test with factors cTBS site (between-subjects factor: visual cortex vs. vertex) and training category (within-subjects factor: trained first vs. trained second). We found a significant interaction between training category and cTBS site (*F*(1,23) = 11.52, *P* = 0.002, *η*^2^ = 0.334; Fig. 2), demonstrating that cTBS altered the pattern of learning on our task. This altered pattern was not modulated by the side of visual training. Indeed, a three-way ANOVA with factors side of visual training (between-subjects factor: left vs. right), training category (within-subjects factor: trained first vs. trained second), and TMS site (between-subjects factor: visual cortex vs. vertex) revealed no significant effects of side of visual training (main effect, two-way interaction with either of the other factors, or a three-way interaction; all *P*’s > 0.1).

To understand the exact nature of the cTBS effects, we compared the performance improvement induced by each of the two training periods. We found a significant difference in performance improvement on the first training between subjects who received cTBS on the visual cortex vs. vertex (*t*(23) = 2.26, *P* = 0.03, Cohen’s *d* = 0.906, independent sample *t*-test, two-sided), such that there was significant performance improvement after cTBS to the vertex (average improvement = 25.8%; *t*(11) = 3.99, *P* = 0.002, Cohen’s *d* = 1.151, one-sample *t*-test, two-sided) but not after cTBS to the visual cortex (average improvement = 5.2%; *t*(12) = 0.82, *P* = 0.43, Cohen’s *d* = 0.227, one-sample *t*-test, two-sided). In other words, cTBS to the visual cortex delivered immediately after the end of the first visual training disrupted the learning associated with this training.

On the other hand, a similar comparison for the second training showed the opposite set of results compared with the first training. We again found a significant difference of cTBS to the visual cortex vs. vertex (*t*(23) = −2.17, *P* = 0.04, Cohen’s *d* = −0.864, independent sample *t*-test, two-sided), but this time in the opposite direction: there was no significant performance improvement after cTBS to vertex (average improvement = 0.4%; *t*(11) = 0.06, *P* = 0.96, Cohen’s *d* = 0.017, one-sample *t*-test, two-sided), but there was now significant performance improvement after cTBS to the visual cortex (average improvement = 18.1%; *t*(12) = 3.9, *P* = 0.002, Cohen’s *d* = 1.081, one-sample *t*-test, two-sided). One possible interpretation of these results is that, by disrupting the learning on the first training, cTBS to the visual cortex released the second training from inhibition.

For completeness, we also directly compared the performance improvement in the first and second training for both the vertex and visual cortex groups. We note that the first and second trainings are not well matched; they differ on multiple variables such as subjects’ prior experience with the task, the time subjects had previously spent in the lab, subjects’ level of tiredness, etc. Therefore, a direct comparison between the performance improvement for each training within a single group can be hard to interpret. Nevertheless, for completeness, we performed such within-group comparisons and found that performance improvement was significantly higher for the first training in the vertex group (*t*(11) = 2.798, *P* = 0.017, Cohen’s *d* = 0.807, paired *t*-test, two-sided) but marginally higher for the second training in the visual cortex group (*t*(12) = −1.873, *P* = 0.085, Cohen’s *d* = 0.519, paired *t*-test, two-sided). Critically, the interaction between these two effects—which controls for the nonspecific factors that are not well matched between the first and the second trainings—was highly significant as reported above (*F*(1,23) = 11.52, *P* = 0.002, *η*^2^ = 0.334).

### Control analyses

We computed the threshold signal-to-noise (S/N) ratio for each orientation by taking the geometric mean of the S/N ratio during the last six reversals in a block as in our previous work^13,25,27,28^. However, to check for the robustness of our findings, we repeated these analyses by taking the geometric mean of the last *k* reversals for *k* from 1 to 8. We found the same pattern of results for all *k*. Specifically, the critical interaction between training category and cTBS site was significant for all *k* (*P* = 0.002, 0.002, 0.001, 0.0004, 0.0006, 0.002, 0.005, and 0.02, respectively, for *k* = 1–8).

**Fig. 3:**
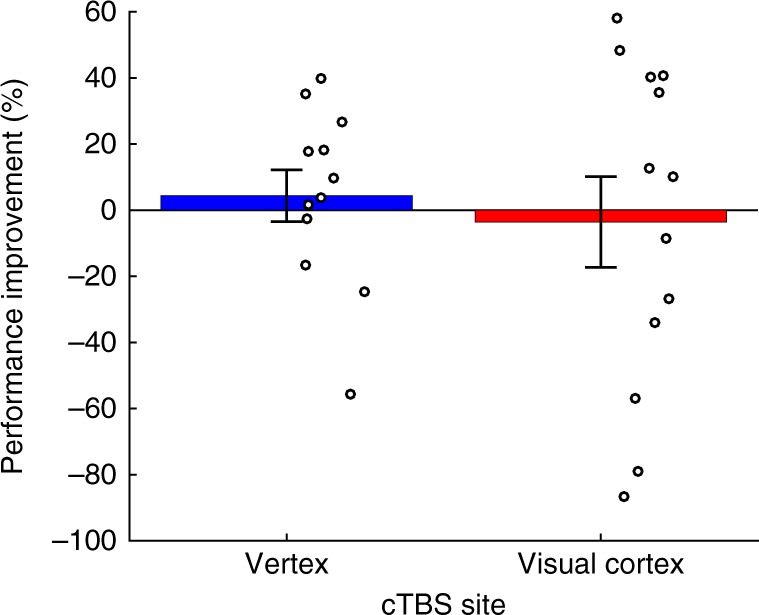
Learning for the untrained orientation. Subjects did not exhibit any performance improvement on the untrained orientation either in the vertex or the visual cortex cTBS conditions. Circles represent individual subjects, error bars represent s.e.m. *N* = 13 for the visual cortex group. *N* = 12 for the vertex group

We also confirmed that our training effects were selective by examining the performance improvement for the untrained Gabor orientation. We found no significant learning for the untrained Gabor orientation in either the subjects who received cTBS to the vertex (average improvement = 4.4%; *t*(11) = 0.56, *P* = 0.59, Cohen’s *d* = 0.161, one-sample *t*-test, two-sided) or the ones who received cTBS to the visual cortex (average improvement = −3.6%; *t*(12) = −0.26, *P* = 0.8, Cohen’s *d* = −0.072, one-sample *t*-test, two-sided). In addition, there was no significant difference between the performance improvement in these two groups for the untrained orientation (*t*(18.899) = 0.502, *P* = 0.62, Cohen’s *d* = 0.199, independent sample t-test, two-sided, violation of equality of variances corrected; Fig. 3).

Finally, we compared the performance improvement for the untrained orientation with the performance improvement for the successful training in each group (first training in the vertex cTBS group and second training in the visual cortex cTBS group). In the vertex group, the performance improvement of first trained orientation was significantly greater than that of the untrained orientation (average improvement of first trained orientation: 25.8%, average improvement of untrained orientation: 4.4%, average difference = 21.4%, *t*(11) = 3.281, *P* = 0.007, Cohen’s *d* = 0.948, paired *t*-test, two-sided). In the visual cortex group, the performance improvement of second trained orientation was numerically greater than that of untrained orientation (average improvement of second trained orientation: 18.1%, average improvement of untrained orientation: −3.6%, average difference = 21.7%). However, even though the size of the effect for the visual cortex cTBS (21.7%) was virtually the same as for the vertex cTBS group (21.4%), the effect in the visual cortex group was not significant (*t*(12) = 1.611, *P* = 0.133, Cohen’s *d* = 0.446, paired *t*-test, two-sided). The lack of significant difference between the secondly trained and the untrained orientations in the visual cortex cTBS group could be due to a number of factors including the relatively high variability in the performance improvement scores in the untrained condition or the second training naturally producing smaller performance improvement compared with the first (e.g., due to subject fatigue). In any case, none of these possible interpretations is at odds with our main conclusions that post training cTBS to the visual cortex abolishes performance improvement and increases the learning generated by subsequent training.

## Discussion

We set to provide causal evidence for the role of the immediate post training period in the consolidation of visual perceptual learning. We applied cTBS to the visual cortex or vertex of human subjects within a few minutes of the training offset. Our results showed that cTBS to the visual cortex abolished performance improvement associated with the training immediately beforehand and released a second training performed 1 h later from inhibition. These findings demonstrate that neural processes within the early visual cortex that occur shortly after the offset of training have a causal role in the consolidation of perceptual learning.

Previous demonstrations of the role of the post training period in the consolidation of new memories have relied primarily on behavioral evidence. Within the domain of motor learning, early studies showed that disrupting the post training neural process by introducing a new motor task abolished performance improvement on the first motor task^5,6^. For example, in a hand-reaching task, subjects can learn to adapt to a specific force field. However, if shortly after learning to adapt to a specific force field subjects need to learn to adapt to a different force field, then no long-term performance improvement is observed anymore for the first force field^5^. Similar results have been found within the visual domain too. Specifically, training on a second task shortly after the first training abolishes performance improvement on the first task for a variety of stimuli^11–13^.

Direct evidence for the causal role of the immediate post training period for learning consolidation has, however, only been demonstrated within motor learning. Several studies applied TMS to the primary motor cortex shortly after training on a simple ballistic finger movement task^7,8^ or sequence learning task^9^. In all cases, TMS abolished the performance improvement on the motor task. This effect was not observed when TMS was delivered to control brain areas or when TMS was applied to the motor cortex 6 h after the practice^8^. These TMS studies demonstrate that the neural processes taking place immediately after the offset of training have a causal effect on the consolidation of motor learning.

A number of previous studies used TMS to understand the mechanisms behind visual perceptual learning^29^. Several studies showed that perceptual learning weakens the interference effects of TMS^30,31^. Other research demonstrated that TMS delivered during the learning phase of a task leads to the disruption of long-term memory formation^32,33^. Finally, more recent studies used TMS to examine the role of different cortical areas in performing a task after extensive visual training and showed changes in the specialization of cortical areas^34,35^.

Although these studies elucidated a number of factors related to visual perceptual learning, none of them examined the role of the immediate post training period. In fact, only one previous study applied TMS in the period after the offset of training in order to disrupt visual perceptual learning. De Weerd et al.^36^ trained subjects on visual stimuli that appeared in either the lower-left or upper-right visual field. The two types of stimuli were trained with no break in-between and TMS was applied 45 min after the offset of the second training. TMS was always delivered to the part of the visual cortex that corresponded to the lower-left location of the visual field. The authors found that TMS abolished learning for the lower-left location when it was trained first (and immediately followed by a training on the upper-right visual field) but not when it was trained second (and preceded by training on the upper-right visual field). De Weerd et al. argued that the effectiveness of TMS only in the first condition was due to high-level areas becoming coupled to the location trained last thus rendering the location trained first vulnerable to disruption by TMS. Our experiment extends these previous results in three important ways. First, we show that TMS applied after a single training can still abolish performance improvement thus demonstrating that the post training period is important even in the absence of competition from two trainings performed immediately after each other. Second, our results revealed that the abolishment of learning via TMS has functional consequences for future learning. Finally, since we applied TMS much earlier after the offset of training (2–3 min) than De Weerd et al. (45 min), our experiment establishes a critical role for the period immediately after training offset in the consolidation of visual perceptual learning.

A critical question in our study is what exact processes were disrupted by the cTBS application. One possibility is that cTBS interfered with processes related to the well-established awake reactivation of recent visual stimuli^37–39^. We previously demonstrated that shortly after visual training on a Gabor orientation, the trained visual feature is spontaneously reactivated in the primary visual cortex^25^. This spontaneous reactivation lasted for at least 8 min but appeared to diminish over time. The strength of the reactivation across subjects was also found to be associated with later learning. Considering that in the current study, we delivered cTBS within 2–3 min after the offset of training, it is highly likely that cTBS disrupted spontaneous awake reactivation in the primary visual cortex. Such disruption could have been caused, for example, by cTBS inducing random noise in the underlying neural population or depressing the activity in the targeted area both of which would prevent the normal course of awake reactivation.

Another possibility is that cTBS disrupted long-term potentiation (LTP) processes related to the first training. Such LTP processes have been hypothesized to be the reason for intertask interference: interference could be due to competition in LTP processes associated with each task^16,40,41^. Indeed, previous studies suggest that cTBS leads to neural inhibition via mechanisms akin to long-term depression^42^ and, within the context of targeting the motor cortex, suppresses the magnitude of the motor evoked potentials in both humans^18,43^ and rats^44^. Therefore, it is possible that cTBS interfered with the LTP processes induced by the first task, which both abolished the performance improvement for this task and released the LTP processes related to the second task from interference.

The two mechanisms described above are nonexclusive and could have both contributed to the effectiveness of cTBS. Future research should combine post-training TMS with imaging techniques in order to investigate its mechanisms more directly.

Another important question is whether the inclusion of the second training was needed for cTBS to the visual cortex to abolish the learning induced by the first training. We favor the interpretation that cTBS would have caused the same abolishment of learning even without the inclusion of the second training. This interpretation is supported by the numerous studies in the motor domain that found post-training TMS abolishing the learning even without the inclusion of a later training^7–9^. Similarly, in the visual domain, as discussed already, de Weerd et al.^36^ found post-training TMS abolishing the previous learning in the absence of further trainings. Nevertheless, it remains possible that within our specific design, the inclusion of the second training was needed in order for cTBS to successfully abolish the learning from the first training. Future studies that do not include a second training are needed to conclusively resolve this issue. Nevertheless, regardless of whether the second training contributed to the cTBS-related abolishment of the first learning, our study provides clear evidence for the role of the post training period in the consolidation of perceptual learning.

In summary, our study suggests that disruption of the immediate post training period abolishes performance improvement on the preceding training and that this abolishment releases later learning from interference. These results provide causal evidence for the notion that post training neural processes within the early visual cortex play a causal role in the consolidation of visual learning. Further, taken together with previous research^7–9^, our findings establish TMS as a technique that can be used to abolish newly formed memories across a variety of domains and raise the possibility of future therapeutic applications.

## Methods

### Subjects

Twenty-five healthy subjects (18–25 years old, 12 females) with normal or corrected-to-normal vision participated in this study. Only subjects with no history of neurological and psychiatric disorders, or any contraindications to TMS and MRI, were allowed to participate. The study was approved by the Institutional Review Board of the Georgia Institute of Technology. All subjects provided written informed consent.

### Procedures

The study consisted of 3 days (Fig. 1). On day 1, subjects who subsequently received stimulation to their visual cortex (*N* = 13) participated in an MRI session. The purpose of the session was to enable us to identify the precise location of stimulation within the early visual cortex. Subjects who received stimulation to a control site (*N* = 12) did not participate in the MRI session. On day 2, all subjects completed three pre training tests and two different training periods on a 2-interval forced-choice (2IFC) orientation detection task (Fig. 1a). The three pre training tests (one block each) were performed for different stimulus orientations (10°, 70°, and 130°), while the trainings (ten blocks each) were performed for two of the three orientations chosen randomly. Immediately after the offset of the first training, subjects received TMS on either early visual cortex or vertex (Fig. 1b). Subjects were given a 1 h break between the two trainings during which they were asked to watch a nature documentary. On day 3, subjects completed a post training test for the same three orientations. Days 1 and 2 were separated by multiple days, while days 2 and 3 were consecutive. The exact interval between days 2 and 3 was 20.5–27.5 h and was comparable between the vertex group (average interval = 22.8 h) and the visual cortex group (average interval = 24.7 h).

### Task

Subjects performed a 2IFC orientation detection task. Each trial began with a 500-ms fixation period. After the fixation period, two stimuli—target and nontarget—were presented for 50 ms each, separated by a 300-ms blank period. Subjects indicated which of the two stimuli contained the target (a Gabor patch) by pressing a button on a keyboard. No feedback was provided after the response. For each subject, the stimulus location was pseudorandomly chosen to be either in the lower-left or lower-right quadrant to avoid any possible hemisphere-specific effects (our analyses show that the side of the stimuli did not affect our results). The center of the stimulus was placed 4° of visual angle away from the center of the screen in a direction of 45° from vertical toward either lower left or lower right. Once the stimulus location was determined for each subject, all visual stimuli were presented only within that quadrant across the whole experiment. The target was a Gabor patch (diameter = 5°, contrast = 100%, spatial frequency = 1 cycle/degree, SD of Gaussian filter = 2.5°, random spatial phase). We varied the S/N ratio by substituting a random selection of pixels from the Gabor patch with noise pixels^13,45,46^. The S/N ratio was defined as the percent of pixels that came from the original Gabor patch. The nontarget stimulus consisted of pure noise (0% S/N ratio). The target interval (first or second) was determined randomly on each trial. During the entire orientation detection task, subjects were required to fixate on a white bullseye (0.75° radius) at the center of the screen. We carefully instructed our subjects to keep fixation but did not use an eye tracker to monitor their eye movements. Nevertheless, in our previous studies using eye tracking, careful instruction resulted in only 1–2% of trials with broken fixation^47^. We also note that breaking fixation should only decrease the effects of the visual cortex cTBS.

The Gabor patches had three possible orientations: 10°, 70°, and 130°. All subjects were tested on all three orientations and received training on two of them. We randomly chose which of the three orientations will be used for the first training, the second training, and which orientation will be untrained.

### Pre- and post-training tests

The pre- and post-training tests were conducted in order to assess the amount of learning that took place. Each pre- and post-training test consisted of three blocks, one for each of the three orientations (10°, 70°, and 130°). The order in which the three orientations were presented during the pre- and post-training tests was determined randomly for each test.

In each pre- and post-training test, we determined the subject-specific threshold intensity for one specific orientation. To do so, we employed a 2-down-1-up staircase procedure as done previously^13,25^. Each block began with 25% S/N ratio and the procedure continuously adjusted the difficulty of the task by altering the S/N ratio. In case the subject’s responses were correct twice in succession (or incorrect once), S/N ratio decreased (increased) with an adaptive step size that varied as a function of the current intensity. To visualize the results of our procedure, we plotted the average S/N as a function of trial number in pre- and post-training tests on days 2 and 3 (Supplementary Fig. 1). Each block terminated after ten reversals. This procedure resulted in subjects completing on average 43 trials per block.

### Training

Subjects were trained on two randomly chosen orientations among the three possible orientations (10°, 70°, and 130°). Each training included only one orientation and consisted of ten blocks. Each training block used the same staircase procedure as in the pre- and post-training tests: it terminated after ten reversals and consisted of about 40 trials. The stimuli were presented on the same side of the screen as during the pre- and post-training tests.

### Analyses

As previously done for this type of task^13,25,27,28^, we calculated the threshold S/N ratio for each orientation by computing the geometric mean of the S/N ratio during the last six reversals in a block. Further, to examine the robustness of the results, we estimated the geometric mean of the S/N ratio during the last *k* reversals for *k* from 1 to 8. Based on the obtained intensity thresholds, we computed the performance improvement from the pre- to the post-training test. For each subject and each orientation, the performance improvement was defined as the percent change in the threshold between pre- and post-training tests:

$$\mathrm{Performance}\;\mathrm{improvement}\;\left( \% \right) = \frac{{T_{\mathrm{pre}} - T_{\mathrm{post}}}}{{T_{\mathrm{pre}}}} \ast 100,$$

where *T*_pre_ and *T*_post_ refer to the threshold S/N ratios before and after training. Note that lower threshold values indicate better performance.

### Statistics and reproducibility

Statistical tests were performed by conducting a two-way mixed measures ANOVA and following up with independent sample and one-sample *t*-tests. For a mixed ANOVA, we tested the assumption of sphericity using Mauchly’s test of sphericity. For independent sample *t*-tests, we also tested if the assumption of equal variances of the dependent variable is met using Levene’s test. The sample size was determined based on similar TMS experiments on visual perceptual learning^34,35^.

### MRI data acquisition

Subjects were scanned in a Siemens 3T Trio MR scanner using a 12-channel head coil. T1-weighted anatomical images were obtained using a multiecho magnetization-prepared rapid gradient echo (MPRAGE; 256 slices, voxel size = 1 × 1 × 1 mm^3^, TR = 2530 ms, FoV = 256 mm).

### Transcranial magnetic stimulation

TMS was delivered using a figure-of-eight magnetic coil (MCF-B65) connected to MagVenture MagPro ×100 Magnetic Stimulator. We employed cTBS^18^, which consists of a series three-pulse bursts at 50 Hz repeated every 200 ms for 40 s (for a total of 600 pulses). Previous studies have shown that the effects of cTBS last for up to an hour after stimulation^18^.

To calibrate the cTBS intensity, we determined the subject-specific motor threshold on day 2 shortly before starting the main experiment using established procedures from our laboratory^48–51^. Briefly, we first applied suprathreshold single pulses around the putative location of the motor cortex in order to identify the best spot for stimulation (defined as the spot that produced maximal finger twitches). We then determined the motor threshold on this location as the minimal TMS intensity required to evoke a visual hand twitch on five of ten consecutive trials. cTBS was then delivered at 80% of the subject-specific motor threshold. Note that this procedure determines the resting motor threshold; other studies employing cTBS (e.g., ref. ^18^) have often used the active motor threshold. Informal interviews after the end of the experiment confirmed that none of the subjects experienced pain or muscle twitches when cTBS was applied to either visual cortex or vertex.

For each subject, we targeted either the vertex or early visual cortex. The vertex served as a control site, whereas the early visual cortex was selected because previous studies suggest that visual learning induces changes in that part of cortex^2,13,19–26^. We note that we did not include a no-TMS control group since vertex TMS is commonly used as the sole control in TMS studies and is not expected to affect visual learning. In cases where we stimulated the vertex, we positioned the TMS coil over Cz with the handle extending posteriorly. In cases where we stimulated the early visual cortex, the coil was positioned over the hot spot marked on an anatomical scan of the subject’s brain with the handle extending dorsally. The hot spot was localized in the calcarine sulcus based on anatomical landmarks so as to correspond to the trained region of the early visual cortex. Subjects who were trained on the lower-left (right) quadrant were stimulated on the corresponding location in the right (left) hemisphere’s dorsal region of the early visual cortex. Stimulation was delivered within 2–3 min after the offset of the first training.

### Apparatus

All stimuli were generated in MATLAB using the Psychtoolbox 3^52^. The visual stimuli were presented on an LCD display (1024  × 768 resolution, 60 Hz refresh rate, Mac OS X) in a quiet, dimly lit room.

### Reporting summary

Further information on research design is available in the Nature Research Reporting Summary linked to this article.

## Supplementary information

Supplementary Information

Reporting Summary

## Data Availability

All raw data have been made freely available via Zenodo^53^ at https://zenodo.org/record/3332947.

## Code availability

All analysis codes have been made freely available via Zenodo^53^ at https://zenodo.org/record/3332947.
