# Supplementary material for: Post-training TMS abolishes performance improvement and releases future learning from interference
Source: Commun Biol. 2019 Aug 27;2:320. doi: 10.1038/s42003-019-0566-4 (PMC6711956; doi:10.1038/s42003-019-0566-4)
Supplement: Supplementary file 1 — Supplementary Information [file 42003_2019_566_MOESM1_ESM.pdf]

Supplementary Figure

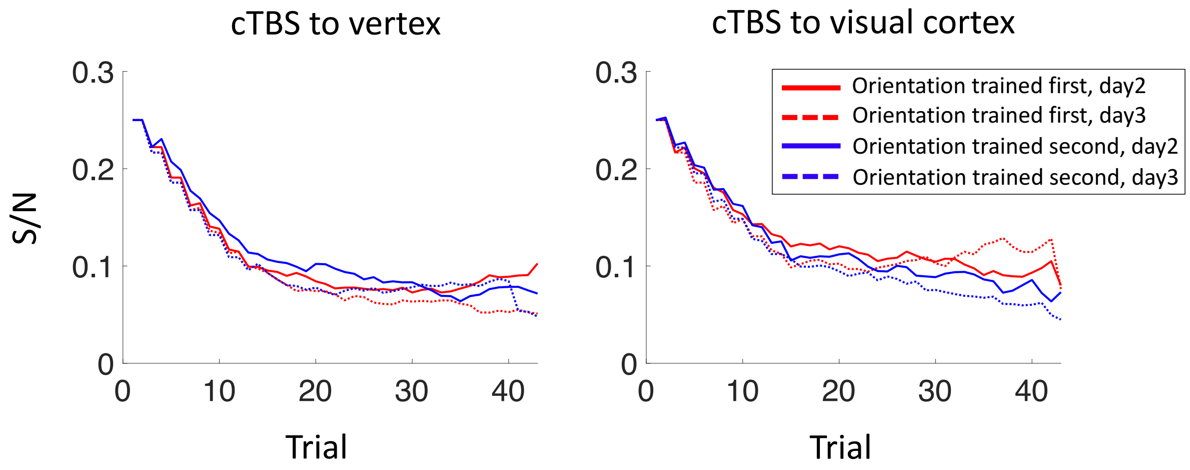

Supplementary Figure 1. Average S/N as a function of trial number in pre- and post-training tests on Days 2 and 3. Each block started with 25% S/N ratio. Different blocks had different number of trials and the average number of trials of pre- and post-training tests was 43. Therefore, in the figure above, we plotted the data from trial 1 to trial 43. N=13 for the visual cortex group. N=12 for the vertex group.
