# Supplementary material for: Post-training TMS abolishes performance improvement and releases future learning from interference
Source: Commun Biol. 2019 Aug 27;2:320. doi: 10.1038/s42003-019-0566-4 (PMC6711956; doi:10.1038/s42003-019-0566-4)
Supplement: Supplementary file 2 — Reporting Summary [file 42003_2019_566_MOESM2_ESM.pdf]

Corresponding author(s): Dobromir Rahnev
Last updated by author(s): Aug 2, 2019

Reporting Summary

Nature Research wishes to improve the reproducibility of the work that we publish. This form provides structure for consistency and transparency in reporting. For further information on Nature Research policies, see Authors & Referees and the Editorial Policy Checklist.

Statistics

For all statistical analyses, confirm that the following items are present in the figure legend, table legend, main text, or Methods section.

n/a	Confirmed	
☐	☒	The exact sample size (*n*) for each experimental group/condition, given as a discrete number and unit of measurement
☐	☒	A statement on whether measurements were taken from distinct samples or whether the same sample was measured repeatedly
☐	☒	The statistical test(s) used AND whether they are one- or two-sided<br>*Only common tests should be described solely by name; describe more complex techniques in the Methods section.*
☒	☐	A description of all covariates tested
☐	☒	A description of any assumptions or corrections, such as tests of normality and adjustment for multiple comparisons
☐	☒	A full description of the statistical parameters including central tendency (e.g. means) or other basic estimates (e.g. regression coefficient) AND variation (e.g. standard deviation) or associated estimates of uncertainty (e.g. confidence intervals)
☐	☒	For null hypothesis testing, the test statistic (e.g. *F*, *t*, *r*) with confidence intervals, effect sizes, degrees of freedom and *P* value noted<br>*Give P values as exact values whenever suitable.*
☒	☐	For Bayesian analysis, information on the choice of priors and Markov chain Monte Carlo settings
☒	☐	For hierarchical and complex designs, identification of the appropriate level for tests and full reporting of outcomes
☐	☒	Estimates of effect sizes (e.g. Cohen's *d*, Pearson's *r*), indicating how they were calculated

Our web collection on statistics for biologists contains articles on many of the points above.

Software and code

Policy information about availability of computer code

Data collection	The data was collected via MATLAB and the Psychtoolbox 3 (Brainard 1997).
Data analysis	The data was analyzed via MATLAB custom code and they are freely available at: https://github.com/DobyRahnev/visual_learning_interference_cTBS.

For manuscripts utilizing custom algorithms or software that are central to the research but not yet described in published literature, software must be made available to editors/reviewers. We strongly encourage code deposition in a community repository (e.g. GitHub). See the Nature Research guidelines for submitting code & software for further information.

Data

Policy information about availability of data

All manuscripts must include a data availability statement. This statement should provide the following information, where applicable:

- Accession codes, unique identifiers, or web links for publicly available datasets
- A list of figures that have associated raw data
- A description of any restrictions on data availability

All raw data and analysis codes are freely available at: https://github.com/DobyRahnev/visual_learning_interference_cTBS.

Field-specific reporting

Please select the one below that is the best fit for your research. If you are not sure, read the appropriate sections before making your selection.

☐ Life sciences ☒ Behavioural & social sciences ☐ Ecological, evolutionary & environmental sciences

Behavioural & social sciences study design

All studies must disclose on these points even when the disclosure is negative.

Study description	Quantitative experimental study.
Research sample	Twenty-five undergraduates (18 to 25 years old, 12 females, 13 males) participated in the study. The sample is representative of the healthy population. The sample size was determined based on similar TMS experiments on visual perceptual learning (Baldassarre et al., 2016; Chen et al., 2016).
Sampling strategy	We used random sampling strategy. The sample size was determined based on similar TMS experiments on visual perceptual learning (Baldassarre et al., 2016; Chen et al., 2016). Previous TMS experiments used comparable sample sizes.
Data collection	The data was collected via MATLAB on Mac computer. During data collection, no one was present besides the subject. The researcher was not blind to experimental condition and the study hypothesis during data collection.
Timing	The data collection started on May 23, 2016 and finished on May 9, 2017. The MRI and behavioral data was collected on the following dates: May 23-24; June 1-2, 7-8, 28-29; July 18-19; August 2-3, 30-31; September 6-7, 12-13, 19-20; October 3-4, 6, 11-13, 19-20, 25-28; November 1-3, 8-10, 15-18, 29-30; December 5; January 18-19; February 21-22; April 11-12; May 5, 8-9
Data exclusions	We did not exclude data.
Non-participation	5 additional subjects declined to participate in the study due to no more interests and time conflicts.
Randomization	Participants were randomly allocated to one among two groups.

Reporting for specific materials, systems and methods

We require information from authors about some types of materials, experimental systems and methods used in many studies. Here, indicate whether each material, system or method listed is relevant to your study. If you are not sure if a list item applies to your research, read the appropriate section before selecting a response.

Materials & experimental systems

n/a	Involved in the study	
☒	☐	Antibodies
☒	☐	Eukaryotic cell lines
☒	☐	Palaeontology
☒	☐	Animals and other organisms
☐	☒	Human research participants
☒	☐	Clinical data

Methods

n/a	Involved in the study	
☒	☐	ChIP-seq
☒	☐	Flow cytometry
☐	☒	MRI-based neuroimaging

Human research participants

Policy information about studies involving human research participants

Population characteristics	See above.
Recruitment	We posted flyers around the university and those who are interested in the study contacted us. No bias occurred in the recruitment process.
Ethics oversight	The study was approved by the Institutional Review Board of Georgia Institute of Technology.

Note that full information on the approval of the study protocol must also be provided in the manuscript.

Magnetic resonance imaging

Experimental design

Design type	NA
Design specifications	NA
Behavioral performance measures	NA

Acquisition

Imaging type(s)	Structural
Field strength	3 Tesla
Sequence & imaging parameters	High-resolution T1-weighted MR images were acquired using a multi-echo magnetization-prepared rapid gradient echo (MPRAGE; 256 slices, voxel size = 1 × 1 × 1 mm3, TR = 2530 ms, FoV = 256 mm)
Area of acquisition	Whole brain scan

Diffusion MRI ☐ Used ☒ Not used

Preprocessing

Preprocessing software	NA
Normalization	NA
Normalization template	NA
Noise and artifact removal	NA
Volume censoring	NA

Statistical modeling & inference

Model type and settings	NA
Effect(s) tested	NA

Specify type of analysis: ☐ Whole brain ☐ ROI-based ☐ Both

Statistic type for inference (See Eklund et al. 2016)	NA
Correction	NA

Models & analysis

n/a	Involved in the study
☒	☐ Functional and/or effective connectivity
☒	☐ Graph analysis
☒	☐ Multivariate modeling or predictive analysis
